# On pyridine chloronium cations†

**DOI:** 10.1039/d2sc06757a

**Published:** 2023-02-06

**Authors:** Patrick Pröhm, Willi Berg, Susanne Margot Rupf, Carsten Müller, Sebastian Riedel

**Affiliations:** a Department of Chemistry and Biochemistry, Freie Universität Berlin Fabeckstr. 34/36 14195 Berlin Germany s.riedel@fu-berlin.de

## Abstract

We present the first solid-state structural evidence of mono- and bis(pyridine)chloronium cations. The latter was synthesized from a mixture of pyridine, elemental chlorine and sodium tetrafluoroborate in propionitrile at low temperatures. The mono(pyridine) chloronium cation was realized with the less reactive pentafluoropyridine, using ClF, AsF_5_, and C_5_F_5_N in anhydrous HF. During the course of this study, we also investigated pyridine dichlorine adducts and found a surprising disproportionation reaction of chlorine that depended on the substitutional pattern of the pyridine. Electron richer dimethylpyridine (lutidine) derivatives favor full disproportionation into a positively and a negatively charged chlorine atom which forms a trichloride monoanion, while unsubstituted pyridine forms a 1 : 1 py·Cl_2_ adduct.

## Abstract 2

The lutidine elephants are pulling apart a dichlorine molecule and stabilizing the corresponding chloronium cation. The remaining chloride forms a trichloride anion. Unsubstituted pyridine, however, only forms a strongly polarized dichlorine adduct.

## Graphical abstract

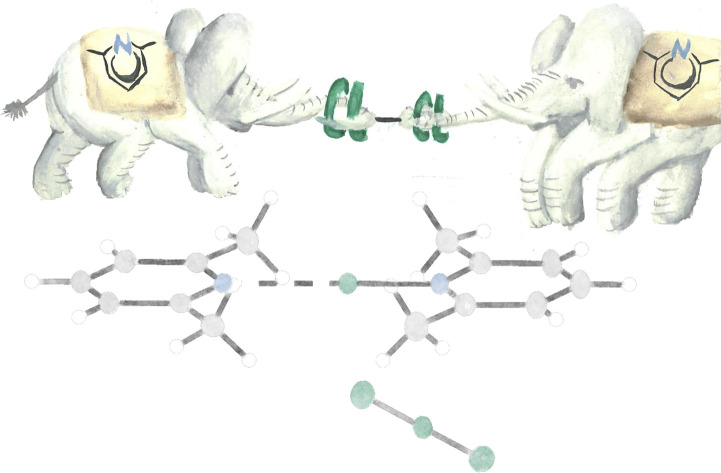

Halonium ions are cations that bear a positive charge at the halogen center.^1^ With nitrogen-based ligands, especially pyridine, of the type [bis(pyridine)X]^+^ or [(pyridine)X]^+^, they are known for all four halogens (Fig. 1). For iodine, bis(pyridine)iodonium(i), also known as Barluenga's reagent,^2–4^ as well as mono(pyridine)iodonium(i) are known.^2,5,6^ Bis(pyridine)iodonium(i) salts and their derivatives are well studied and have manifold applications in organic chemistry as halogenation or oxidation reagents.^4,7^ Additionally, it acts as a model system for the systematic study of halogen bonding as numerous studies show.^8^ The [(pyridine)I]^+^ cation is characterized by means of IR (ref. 5) and NMR (ref. 6) spectroscopy as well as elemental analysis.^5^ Regarding the lighter homologue bromine only the bis(pyridine)bromonium(i) and its derivatives are known and used as a reagent in organic chemistry, similar to the iodine species.^9^ For the lightest halogen fluorine, the *N*-fluoropyridinium cation is known and widely applied as an electrophilic fluorination reagent.^10,11^ Coordination of a second pyridine towards the fluorine atom is only weak and, in contrast to the heavier bis(pyridine)halonium(i) cations, asymmetric coordination with one long and one short N–F bond is preferred, due to the inverse polarity of the bond.^12,13^ Chloronium cations with pyridine ligands have not been structurally characterized in the solid-state, yet. However, the bis(pyridine)chloronium(i) cation was investigated in solution by NMR spectroscopy, regarding its 3c–4e bonding nature as well as in the gas-phase by mass spectrometry.^12,14,15^ The mono(pyridine)chloronium(i) cation was previously detected in mass spectrometric studies.^14,15^ Additionally, the mono- and bis(pyridine)chloronium(i) cations were studied in several different computational studies.^16^ In this context, the Dutton group conducted a highly elucidating theoretical study on their thermodynamic stabilities, dissociation pathways, and overall bonding nature.^13^ Apart from the cationic species with a single halogen atom mentioned above, complexes between pyridine and dihalogens of the type pyridine·X_2_ are known for all four halogens (X = F, Cl, Br, I). In cases of the two heavier halogens bromine and iodine, the complexes are thoroughly studied by vibrational and NMR spectroscopic data.^13,17,18^ In case of the pyridine·I_2_ complex a solid-state structure is reported.^19^ Additionally, pyridine·Br_2_ can be used as a bromination reagent for aromatic substrates.^20^ The lighter congener pyridine·Cl_2_ is also used as a reagent for the oxidation of secondary alcohols to the corresponding carbonyls.^21^ However, the reagent is generated *in situ* and was never isolated or spectroscopically described. The complex between pyridine and molecular fluorine is known and can be isolated. However, it violently decomposes at temperatures above −2 °C.^22^ Therefore, it is not surprising that only a marginal amount of analytical data is known.^10,11^

**Fig. 1:**
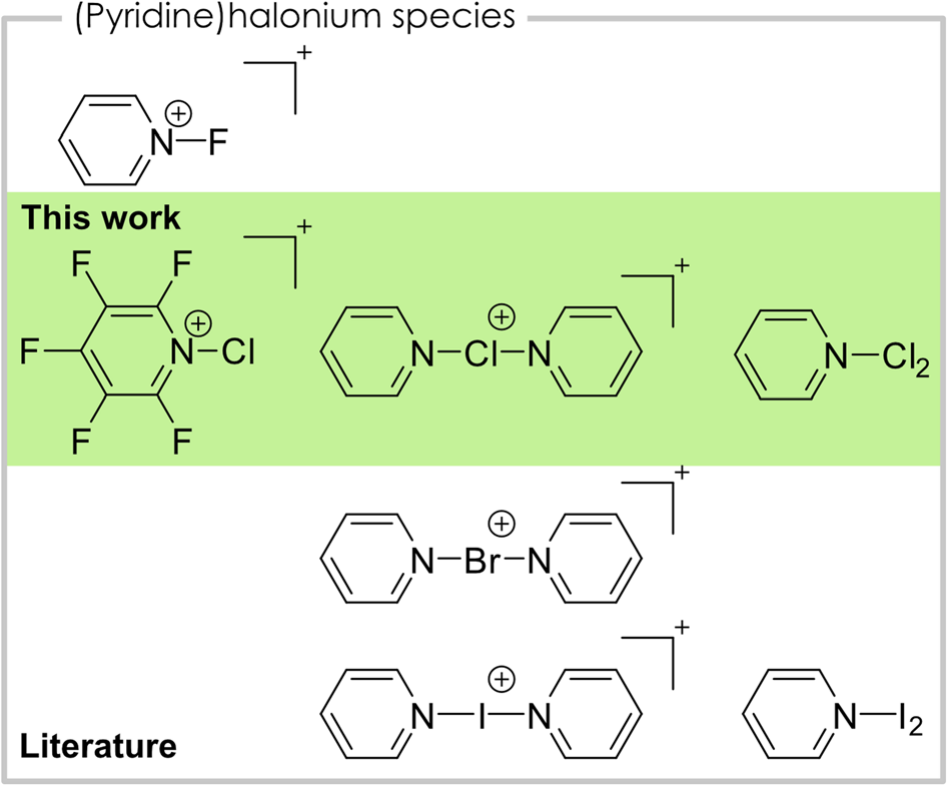
Overview of pyridine-halonium compounds structurally characterized in the solid-state.

We report the synthesis of a series of chloronium ions with different pyridine-based ligands. The pyridine chlorine complex pyrinde·Cl_2_ is readily synthesized from equimolar amounts of pyridine and chlorine in propionitrile, acetonitrile, dichloromethane, or *ortho*-difluorobenzene at −40 °C which results in a colorless precipitate. Although used as an oxidizing reagent this complex has basically not been studied. Single crystals were obtained at −80 °C from a propionitrile solution. Fig. 2 (left) shows the corresponding solid-state structure. The Cl1–Cl2 distance is 266.23(6) pm and the N–Cl1 distance has a bond length of 175.4(2) pm. It can be interpreted either as a neutral complex between pyridine and chlorine (pyridine·Cl_2_), with a weak Cl–Cl bond, or alternatively as a pyridine chloronium cation [(pyridine)Cl]^+^, which is strongly interacting with its chloride counterion. We calculated the natural charges (NPA) based on the atomic coordinates of the crystal structure and found +0.18 a.u. for the central chlorine atom, while the terminal chlorine atom bears a charge of −0.62 a.u. and the nitrogen atom of −0.29 a.u. Due to this charge distribution we conclude the compound has significant ionic contributions and can be interpreted as [(pyridine)Cl]^+^Cl^−^, similar to [(pyridine)F]^+^F^−^.^22^ Additionally, we optimized (B3LYP/PCM(EtCN)/def2-TZVPP) the structure but even with a solvation model representing propionitrile the bond lengths are poorly reproduced (*d*_calc_(Cl–Cl) = 231.4 pm, *d*_calc_(N–Cl) = 201.8 pm) with more than 25 pm discrepancy for both bonds. Computing the cation [(pyridine)Cl]^+^ alone, the structural parameters match relatively well with a calculated Cl–N bond distance of 171.1 pm. The crystal packing is dominated by halogen bonding interactions between [(pyridine)Cl]^+^ and Cl^−^ as evident from the almost linear arrangement of the Cl–Cl–N fragment (Cl2–Cl1–N = 178.02(4)°), which can be rationalized by the σ-hole along the N–Cl1 bonding axis. Additionally, the negatively polarized chlorine atom forms a hydrogen bond with the H atom in 4-position of a neighboring pyridine ring (Fig. S2†). The layered structure of the compound can be rationalized by anion–π interactions between the negatively polarized chlorine atom and the pyridine ring. This chlorine atom is located above a heterocycle of the next plane with a distance of 327.97(2) pm between the Cl atom and the plane (Fig. S2†). The Raman spectrum (Fig. S5†) is dominated by a double band at *ṽ* = 360 and 345 cm^−1^ with a shoulder at *ṽ* = 335 cm^−1^, which are attributed to the N–Cl stretching vibrations according to periodic solid-state calculations.

**Fig. 2:**
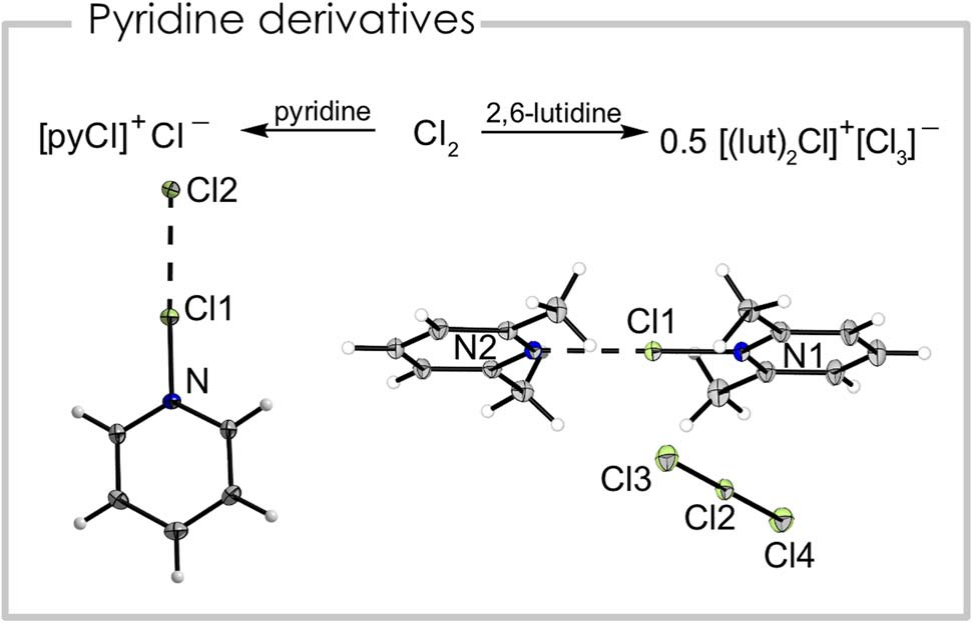
Left: synthetic route and solid-state structure of [(pyridine)Cl]Cl. Selected bond distances (pm) and angles (°): N–Cl1 = 175.4(2), Cl1–Cl2 = 266.23(6), Cl2–Cl1–N = 178.02(4). Right: synthetic route and solid-state structure of [(2,6-lutidine)_2_Cl][Cl_3_]. Selected bond distances (pm) and angles (°): Cl1–N1 177.9(1), Cl1–N2 240.0(1), Cl2–Cl3 231.22(7), Cl2–Cl4 225.48(7), N1–Cl1–N2 179.95(6), Cl3–Cl2–Cl4 179.74(3). Color code: green = chlorine, blue = nitrogen, grey = carbon, white = hydrogen.

When replacing the base pyridine with the more electron-rich 2,6-lutidine (2,6-dimethylpyridine) we observed a different reactivity with equimolar amounts of chlorine. Instead of the analogous 2,6-lutidine·Cl_2_ adduct we found the disproportionation of chlorine into a bis(2,6-lutidine)chloronium(i) cation and trichloride anion, maintaining the formal 1 : 1 stoichiometry between the heterocycle and Cl_2_ (Fig. 2 right). Note, that here a negatively charged trichloride anion is stabilized by a positively charged chloronium cation. We were able to grow a single crystal suitable for X-ray diffraction of the bis(2,6-lutidine)chloronium(i) trichloride. The compound crystallized in the monoclinic space group *P*2_1_/*m*. Interestingly, the two nitrogen chlorine distances are not equal and exhibit bond lengths of 177.9(1) pm and 234.0(1) pm. The N–Cl–N bonding angle is essentially linear (179.95(6)°) and the two 2,6-lutidine rings are in the same plane. The trichloride anion also has two different bond lengths (231.22(7), 255.48(7) pm).^23^ The Raman spectrum (Fig. S8†) shows a strong band at *ṽ* = 278 cm^−1^, which is attributed to the trichloride anion, in agreement with other trichloride anions.^24^ We assigned the band at *ṽ* = 375 cm^−1^ to the N–Cl stretching mode according to quantum-chemical calculations. Note, that for the bromine pyridine system the solvent dependent equilibrium between [bis(pyridine)Br][Br_3_] and pyridine·Br_2_ is known.^18^

We were able to generate a [bis(pyridine)Cl]^+^ cation from the reaction of two equivalents of pyridine with one equivalent of chlorine and sodium tetrafluoridoborate, under precipitation of sodium chloride (Fig. 3). Single crystals suitable for X-ray diffraction were obtained by slowly cooling a propionitrile solution containing [bis(pyridine)Cl][BF_4_] to −80 °C. The asymmetric unit consists of three cationic fragments, two half occupied and one fully occupied, two tetrafluoridoborate anions and two solvent molecules (EtCN). All three crystallographically inequivalent [bis(pyridine)Cl]^+^ cations have a shorter (179.3(5) to 188.5(4) pm) and a longer N–Cl bond (208.5(4) to 223.2(6) pm, details see Table 1). This minor asymmetry is known from its heavier analogues [bis(pyridine)Br]^+^ and [bis(pyridine)I]^+^ and is likely a result of crystal packing effects.^25^ The N–Cl–N angles are linear or close to linear (178.8(2)°) and the heterocycles are slightly twisted (3.9(2)° to 17.6(3)°). The calculated structure (B3LYP/def2-TZVPP) however has, in agreement with literature studies (*vide supra*),^12^ two identical bond lengths (200.3 pm), since it is optimized in pseudo-gas-phase environment neglecting crystal lattice interactions, that are most likely responsible for the minor asymmetry. Consequently, we experimentally observe two Raman bands (Fig. S6†) for the N–Cl vibration at *ṽ* = 285 and 116 cm^−1^ in comparison to one calculated band at *ṽ* = 177 cm^−1^. We studied the dependency of the ^1^H NMR shifts of the pyridine moieties at low temperatures (Fig. S12†) and found a downfield shift for all three signals in [(pyridine)Cl]Cl with respect to pyridine under identical conditions (Δ*δ*(^1^H_*ortho*_) = 0.06, Δ*δ*(^1^H_*meta*_) = 0.32, Δ*δ*(^1^H_*para*_) = 0.36 ppm). The ^1^H NMR signals of the [bis(pyridine)Cl]^+^ cation showed a similar downfield shift (Δ*δ*(^1^H_*ortho*_) = 0.09, Δ*δ*(^1^H_*meta*_) = 0.33, Δ*δ*(^1^H_*para*_) = 0.36 ppm) which is in agreement with literature values.^12^

**Fig. 3:**
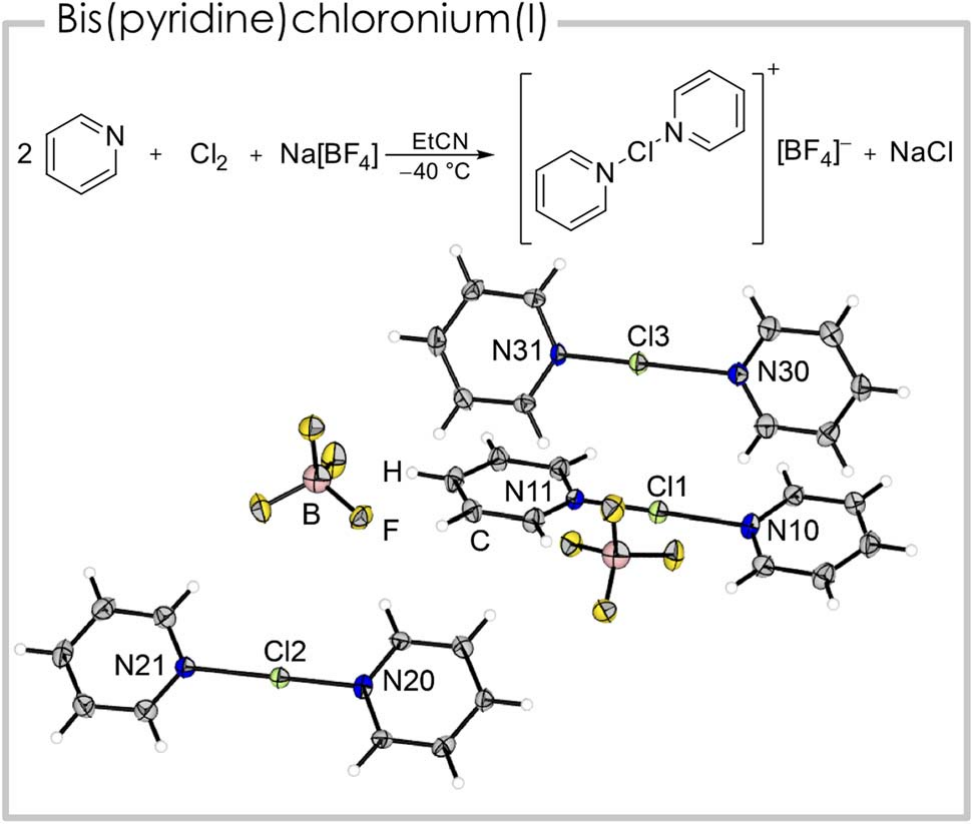
Synthetic route and solid-state structure of [bis(pyridine)Cl][BF_4_]. Solvent omitted for clarity. Color code: green = chlorine, blue = nitrogen, grey = carbon, white = hydrogen, yellow = fluorine, rose = boron. Selected bond distances (pm) and angles (°): Cl1–N10 208.5(4), Cl1–N11 188.4(4), Cl2–N20 186.3(5), Cl2–N21 211.3(6), Cl3–N30 223.2(6), Cl3–N31 179.3(5).

**Table 1:** Chlorine–Nitrogen bond lengths of different pyridine chloronium compounds

Compound	Bond	*d*/pm^a^	Σ*d*/pm
[bis(pyridine)Cl][BF_4_]	Cl1–N10	208.5(4), *199.9*	396.9
	Cl1–N11	188.4(4)	
	Cl2–N20	186.3(5)	397.6
	Cl2–N21	211.3(6)	
	Cl3–N30	223.2(6)	402.5
	Cl3–N31	179.3(5)	
[(pyridine)Cl]Cl	Cl–N	175.4(2), *193.1*^b^	—
	Cl–N	177.7(2)	—
[C_5_F_5_NCl][AsF_6_]	Cl–N	169.6(3), *170.0*	—

a *Italic*: calculated (B3LYP/PCM(EtCN)/def2-TZVPP) bond lengths of the cationic moiety.

b Solid state calculation (see Computational details).

If silver tetrafluoridoborate instead of sodium tetrafluoridoborate is used for precipitation of the chloride anion, the reaction yields two products. The first is the previously described [bis(pyridine)Cl][BF_4_]. The other is the Ag^II^-containing compound [Ag(pyridine)_4_][BF_4_]_2_. This complex has been known since over a century and has been extensively studied by EPR spectroscopy,^26^ however, a solid-state structure is so far unknown. Here, we report the solid-state structure of the [Ag(pyridine)_4_][BF_4_]_2_ complex (Fig. S4,† EPR Fig. S13†). Overall, the finding of this complex is quite unexpected due to the high redox potential of Ag^II^ compounds ([Ag(2,2′-bipyridine)_2_][ClO_4_]_2_: *E*^NHE^ = 1.45 V,^27^*cf*. Cl_2_*E*^NHE^ = 1.396 V (ref. 28)). From this result, we conclude that the oxidation potential of chlorine has been enhanced in the reaction medium. We hypothesize that the reactive species is a mono(pyridine)chloronium(i) cation [(pyridine)Cl]^+^ that was generated *in situ* from [(pyridine)Cl]Cl and Ag[BF_4_] after precipitation of the negatively polarized chlorine atom as AgCl. An attempted generation of this reactive intermediate by combination of [(pyridine)Cl]Cl with Lewis acids such as BCl_3_, GaCl_3_ or B(C_6_F_5_)_3_ yielded multicomponent mixtures even at temperatures below −40 °C, indicating the formation of a reactive intermediate. Generally, activation of dihalogens has been accomplished before by usage of Ag^I^ salts that form coordination complexes of the type [Ag(X_2_)]^+^.^29^ For the isolation of such weakly bound complexes, weakly coordinating anions (WCAs) such as [Al(OC(CF_3_)_3_)_4_]^−^ were used.

The synthesis of a mono(pentafluoropyridine)chloronium(i) cation was achieved from the reaction of pentafluoropyridine with [Cl_2_F][AsF_6_]. The latter is a strong chlorination reagent and is synthesized from two equivalents of ClF and one equivalent of AsF_5_ in anhydrous HF (*a*HF).^30^ Despite being well-characterized spectroscopically, a crystal structure was missing up to now, probably due to the cation's disproportionation behavior into [Cl_3_]^+^ and [ClF_2_]^+^.^31^ However, we were able to grow single-crystals suitable for X-ray diffraction (see Fig. 4 top and Fig. S3†) from an *a*HF solution and prove the suspected asymmetric, bent structure of the triatomic cation.^31^ The Cl–Cl bond length is 193.87(8) pm and the Cl–F bond length is 159.4(2) pm. The bond angle in the cation is 101.83(7)°.

**Fig. 4:**
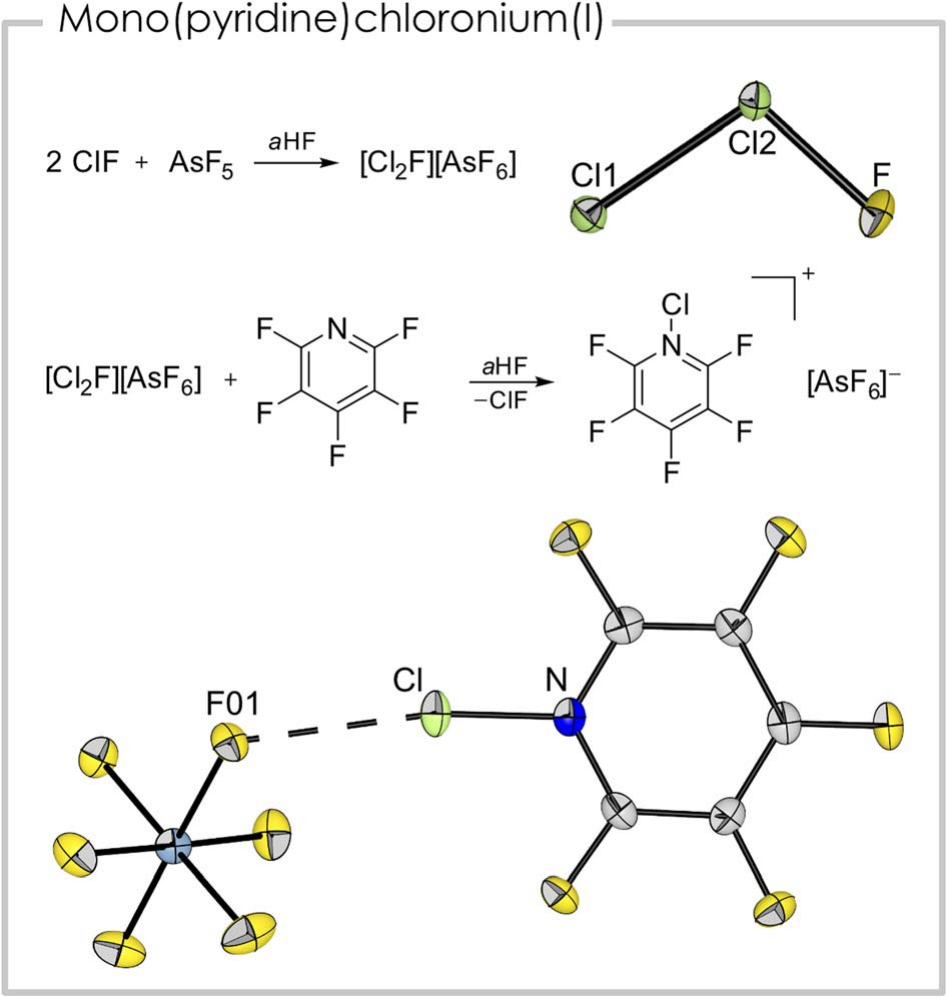
Top: synthetic route and solid-state structure of [Cl_2_F][AsF_6_] (anion omitted) selected bond distances (pm) and angles (°): Cl1–Cl2 193.87(8), Cl2–F 159.4(2), Cl1–Cl2–F 101.83(7) bottom: synthetic route and solid-state structure of [(C_5_F_5_N)Cl][AsF_6_]. Color code: green = chlorine, blue = nitrogen, grey = carbon, yellow = fluorine, light blue = arsenic. Selected bond distances (pm) and angles (°): Cl–N 169.6(3), Cl–F01 263.7(2), N–Cl–F01 170.5(1).

The addition of one equivalent of pentafluoropyridine to an *a*HF solution containing [Cl_2_F][AsF_6_] led to [C_5_F_5_NCl][AsF_6_] (Fig. 4 bottom). Notably, it is the first report of a mono(pyridine)chloronium cation. The compound crystallized in the space group *P*2_1_/*c*. The length of the nitrogen chlorine bond (169.6(3) pm) is the shortest amongst the here presented (pyridine)chloronium ions. This is surprising since the basicity of pentafluoropyridine is significantly weaker than the basicity of the non-fluorinated derivative. The shortest cation–anion contact is between F01 and Cl (263.7(2) pm) and is well below the sum of the van der Waals radii (∑*r*^vdW^(Cl–F) = 322 pm (ref. 32)). Together with the N–Cl–F01 bond angle of 170.5(1)°, this interaction is likely a halogen bond. In contrast to the [bis(pyridine)Cl]^+^ entities, which possess 3c–4e character,^25^ this interaction is probably more of electrostatic nature, indicated by the pronounced asymmetry of the [F–Cl–N] moiety (*c.f.* Cl–N 169.6(3), Cl–F01 263.7(2) pm). Nevertheless, this weak coordination appears to stabilize the highly illusive cation. Apart from the aforementioned interaction, further cation–anion contacts are found in the solid-state structure. These are mainly anion–π interactions^33^ of the fluorine atoms of two [AsF_6_]^−^ anions located above and below the cation ring plane. The distances between the cation plane and the anion related F atoms are between 286.0(4) pm (F10-plane) and 288.4(5) pm (F14-plane, Fig. S1†). The shortest distance between the centroid of the ring and an anion-related F atom is 295.5(3) pm (F11-ring centroid). The experimentally determined bond lengths are well reproduced in the gas-phase calculation (B3LYP/PCM(EtCN)/def2-TZVPP; *d*_calc_(N–Cl) = 170.0 pm). The NPA of the gas-phase optimized structure revealed a positive charge of +0.34 a.u. at the chlorine atom and a negative charge of −0.34 a.u. for the nitrogen atom. The measured Raman spectrum (Fig. S7†) exhibits a band at *ṽ* = 603 cm^−1^, which has contribution of the N–Cl stretching mode together with a breathing mode of the heterocycle, according to quantum-chemical calculations.

In conclusion, we presented the first structural evidence of different (pyridine)chloronium(i) cations. Pyridine and chlorine form a strong adduct that has a significant ionic character. It is a potent oxidizer and can oxidize Ag^I^ to Ag^II^. The bis(pyridine)chloronium(i) cation is synthesized from pyridine, chlorine, and sodium tetrafluoridoborate. Increasing the basicity of the pyridine moiety by introducing two methyl groups (2,6-lutidine) leads to the full disproportionation of dichlorine, forming a bis(2,6-lutidine)chloronium(i) cation and a trichloride anion. For the synthesis of the first mono(pyridine)chloronium(i) cation, we used pentafluoropyridine and the strong chlorination reagent [Cl_2_F][AsF_6_], which was structurally characterized for the first time.

**Caution!** Chlorine monofluoride and arsenic pentafluoride are extraordinarily reactive and can react violently with organic materials under formation of hydrogen fluoride. Hydrogen fluoride is extremely toxic and reacts with glass.

## Author contributions

P. P. designed the project, performed experiments and gas-phase calculations, collected, and interpreted spectroscopic data and wrote the manuscript. W. B. performed experiments and collected spectroscopic data. S. M. R. collected XRD data and solved and refined the crystal structures. C. M. performed solid-state calculations. All authors discussed and commented on the manuscript. S. R. directed and coordinated the research.

## Conflicts of interest

There are no conflicts to declare.

## Supplementary Material

SC-014-D2SC06757A-s001

SC-014-D2SC06757A-s002
