# Supplementary material for: On pyridine chloronium cations
Source: Chem Sci. 2023 Feb 6;14(9):2325–9. doi: 10.1039/d2sc06757a (PMC9977394; doi:10.1039/d2sc06757a)
Supplement: SC-014-D2SC06757A-s001 [file SC-014-D2SC06757A-s001.pdf]

Electronic Supplementary Material (ESI) for Chemical Science.
This journal is © The Royal Society of Chemistry 2023

ESI

General Information

All experiments were performed under rigorous exclusion of moisture and oxygen using Schlenk techniques. For precise stoichiometries, chlorine was condensed and weighed in 5 ml pressure resistant glass tubes equipped with Teflon screw stoppers by Rettberg. The amount of pyridine was adjusted according to the amount of chlorine used. The reaction itself was carried out in standard Schlenk flasks or tubes. Crystallization was accomplished by storing a Schlenk tube with a concentrated solution at −40 °C in a large Dewar filled with −40 °C ethanol and placing the Dewar in a−80 °C freezer. Solid material was isolated by filtration through a cannula equipped with a glass filter paper and Teflon tape. Solids were handled in a dry box under argon atmosphere (O_2 < 0.5 ppm, H_2O < 0.5 ppm). ClF, *a*HF and AsF_5 were handled in a well passivated (3 bar F_2) stainless steel line equipped with Swagelock and Fitok valves. Propionitrile was dried over Sicapent® prior to use and stored over molecular sieve 3 Å. Pyridine, 2,6-lutidine was dried over CaH_2 and distilled. *a*HF was dried multiple times with elemental fluorine. All other chemicals were used as purchased. ClF[1] was synthesized as described elsewhere. Raman spectra were recorded on a Bruker MultiRAM II equipped with a low-temperature Ge detector (1064 nm, 30-80 mW, resolution 4 cm^{-1}). Spectra of single crystals were recorded at −196 °C using the Bruker RamanScope III using a previously published setup.[2] IR spectra were recorded on a Nicolet iS50 Advance FT-IR by Thermo Fisher Scientific equipped with an ATR unit, with a Ge on KBr beamsplitter and a DLaTGS-KBr detector for MIR and a solid-substrate beamsplitter with a DLaTGS-PE detector for FIR. For low-temperature measurements a previously published setup was used.[3] NMR spectra were recorded on a JEOL 400 MHz ECS or ECZ spectrometer. All reported chemical shifts are referenced to the Ξ values given in IUPAC recommendations of 2008 using the ^{2}H signal of the deuterated solvent as internal reference.[4] For external locking acetone-d6 was flame sealed in a glass capillary and the lock oscillator frequency was adjusted to give δ(^{1}H) = 7.26 ppm for a $CHCl_3$ sample locked on the capillary. EPR spectra were recorded in the X-band at 298 K with a Magnetech MS 5000 spectrometer. Simulations were done with Easyspin. Crystal data were collected on a Bruker D8 Venture diffractometer with a Photon 100 CMOS area detector with MoK_α radiation. Single crystals were picked at –80 °C under nitrogen atmosphere and mounted on a 0.15 mm Mitegen micromount using perfluoroether oil diluted with perfluorohexane. The structures were solved with the ShelXT[5] structure solution program using intrinsic phasing and refined with the ShelXL[6] refinement package using least squares minimizations by using OLEX2.4[7] For visualization the Diamond V3.0 program was used.[8] For structure optimizations of isolated molecules the Gaussian program[9] was used with the DFT hybrid functional B3LYP[10] and the triple-ζ basis set def2-TZVPP.[11] The PCM solvent model with a dielectric constant of acetonitrile was used.[12] As a validation for minimum structures, harmonic frequencies were calculated as implemented. NBO analyses were performed with NBO 7.0.[13] The periodic calculation of the [pyCl]Cl system were performed with the CRYSTAL17[14] program, using the B3LYP DFT functional and employing the Gaussian-type atomic basis set cc-pVDZ.[15] The first Brillouin zone was sampled using an 6×6×6 Monkhorst-Pack grid. To facilitate convergence, the Coulomb and exchange integral thresholds were sufficiently tightened with the TOLINTEG keyword to values of 8, 8, 8, 8 and 16.

Experimental section:

[pyCl]Cl

Pyridine (84 mg, 1.07 mmol, 1 eq) was dissolved in propionitrile. Chlorine gas (76 mg, 1.07 mmol, 1 eq) was added at −196 °C and the reaction mixture was allowed to warm to −40 °C under stirring. After 5 min a colorless solution with a colorless precipitate had formed. For crystallizations the solution was filtered and slowly cooled to −80 °C. The precipitate was spectroscopically identical to the single crystals. The compound decomposed when warming it above −10 °C.

^{1}H NMR (400 MHz, MeCN-d3, 253 K) δ / ppm = 8.62 (d, $^3J(^1H^1H)$ = 5.5 Hz, 2H), 8.26 – 7.94 (m, 1H), 7.65 (t, $^3J(^1H^1H)$ = 6.7 Hz, 1H).

Raman (solid, 1064 nm, 77 K) $\tilde{\nu}$ /cm^{-1}= 3090, 3080, 1610, 1581, 1176, 1069, 1022, 782, 693, 669, 642, 360, 345, 136, 121.

IR (solid, ATR, 193 K) $\tilde{\nu}$ /cm^{-1}= 3097, 3076, 3051, 3035, 3028, 2993, 2059, 1917, 1607, 1588, 1576, 1559, 1534, 1463, 1437, 1430, 1411, 1331, 1322, 1263, 1181, 1088, 1063, 1038, 759, 663, 449, 341, 333, 247, 203, 125.

[py_2Cl][BF_4]

Sodium tetrafluoridoborate (189 mg, 1.72 mmol, 1 eq) and pyridine (277 µl, 3.44 mmol, 2 eq) were dissolved in propionitrile (5 ml). Chlorine (122 mg, 1.72 mmol, 1 eq) was condensed onto the reaction mixture. The reaction mixture was allowed to warm to −40 °C and stirred for 5 h at −40 °C. The reaction mixture was filtered at −40 °C and slowly cooled to −80 °C to obtain single crystals. The compound decomposed when warming it above −10 °C.

^1H NMR (400 MHz, MeCN-d3, 253 K) δ / ppm = 8.65 (d, $^3J(^1H^1H)$ = 5.4 Hz, 4H), 8.09 (td, $^3J(^1H^1H)$ = 7.5, $^4J(^1H^1H)$ = 2.1 Hz, 2H), 7.70 – 7.62 (m, 4H), *c.f.* ref. 16

Raman (solid, 1064 nm, 77 K) $\tilde{\nu}$ /cm^{-1}= 3115, 3097, 3086, 1601, 1578, 1487, 1225, 1032,1024, 782, 756, 668, 655, 644, 289, 130.

IR (solid, ATR, 193 K) $\tilde{\nu}$ /cm^{-1}= 3110, 3084, 3043, 1599, 1464, 1357, 1287, 1264, 1207, 1164, 1054, 1028, 1008, 761, 697, 681, 654, 635, 520, 463.

[$Ag(py)_4$][BF_4]$_2$

Pyridine (292 mg, 4.06 mmol, 4 eq) was dissolved in propionitrile (2.5 ml). Chlorine gas (72 mg, 1.01 mmol, 1 eq) was added at −196 °C and the reaction mixture was allowed to warm to −40 °C and stirred for 5 min. A solution of Ag[BF_4] (395.2 mg, 2.03 mmol, 2 eq) in propionitrile (3.5 ml) was added at −40 °C and the reaction solution was stirred for 30 min at −40 °C. For crystallizations, the orange solution was filtered and slowly cooled to −80 °C.

Raman (crystal, 1064 nm, 77K) $\tilde{\nu}$ /cm^{-1}= 3106, 3094, 3082, 1604, 1574, 1495, 1231, 1044, 1032, 1020, 810, 767, 644, 627, 542, 517, 196, 177, 139, 110

EPR (solid, X-band, 298 K) g_x = 2.035, g_y = 2.081, g_z = 2.130, *c.f.* ref.17

[$(2,6\text{-lutidine})_2Cl$][$Cl_3$]

2,6-Lutidin (118 mg, 1.1 mmol, 1 eq) was dissolved in propionitrile (2 ml). Chlorine gas (78 mg, 1.1 mmol, 1 eq) was added at −196 °C and the reaction mixture was allowed to warm to −40 °C under stirring. After 5 min a colorless solution with a colorless precipitate had formed. For crystallizations the solution was filtered and slowly cooled to −80 °C. The precipitate was spectroscopically identical to the single crystals. The compound decomposed when warming it above −10 °C.

^1H NMR (400 MHz, MeCN-d3, 253 K) δ / ppm = 8.10 (s, 2H), 7.65 (s, 4H), 2.79 (s, 12H).

Raman (crystal, 1064 nm, 77 K) $\tilde{\nu}$ /cm^{-1}= 3074, 3060, 1610, 1578, 1420, 1378, 1281, 1269, 1167, 1147, 1035, 996, 711, 591, 545, 375, 278, 228, 110, 86.

IR (solid, ATR, 193 K) $\tilde{\nu}$ /cm^{-1}= 3089, 3076, 3017, 2907, 1605, 1592, 1578, 1566, 1480, 1375, 1275, 1220, 1183, 1120, 1048, 1028, 798, 735, 702, 589, 464, 357, 289, 268.

[Cl_2F][AsF_6]

Synthesis according to literature.[18] In a PFA tube with 8 mm diameter AsF_5 (1.75 mmol, 1 eq) was dissolved in *a*HF (2 ml) and ClF (3.5 mmol, 2 eq) was added at −196 °C. The reaction mixture was allowed to warm to −40 °C. Single crystals were obtained by slowly cooling a sample in *a*HF to −80 °C. The compound decomposed when warming it above −40 °C.

Raman (solid, 1064 nm, 77K) $\tilde{\nu}$ /cm^{-1} = 807, 744, 685, 583, 532, 376, 295.

[C_5F_5NCl][AsF_6]

In a PFA tube with 8 mm diameter AsF_5 (96.8 µmol, 1 eq) was dissolved in *a*HF (1 ml) and ClF (193.6 µmol, 2 eq) was added at −196 °C. The reaction mixture was allowed to warm to −40 °C. C_5F_5N (10.6 µl, 96.8 µmol, 1 eq) was added at −196 °C and the reaction mixture was allowed to warm to −40 °C. The solvent was removed at −40 °C. Single crystals were obtained by slowly cooling a sample in *a*HF to −80 °C. The compound decomposed when warming it above −40 °C.

Raman (solid, 1064 nm, 77K) $\tilde{\nu}$ /cm^{-1} = 811, 752, 690, 675, 603, 524, 408, 375, 335, 93.

Crystallographic section:

Compound	[pyCl]Cl	$[py_2Cl][BF_4]$
Identification code	Pnma	c2_twin
Empirical formula	$C_5H_5Cl_2N$	$C_{13}H_{15}BClF_4N_3$
Formula weight	150.00	335.54
Temperature/K	100.0	100.0
Crystal system	orthorhombic	monoclinic
Space group	*Pnma*	*C*2
a/Å	13.9435(6)	14.9392(9)
b/Å	6.5593(3)	14.4032(9)
c/Å	7.0669(4)	15.6990(10)
α/°	90	90
β/°	90	108.895(3)
γ/°	90	90
Volume/Å^3	646.34(5)	3196.0(3)
Z	4	8
ρ_{calc}g/cm^3	1.541	1.395
μ/mm^{-1}	0.889	0.277
F(000)	304.0	1376.0
Crystal size/mm^3	0.1 × 0.1 × 0.1	0.26 × 0.25 × 0.19
Radiation	MoK_α (λ = 0.71073)	MoK_α (λ = 0.71073)
2Θ range for data collection/°	5.844 to 61.046	4.038 to 52.814
Index ranges	-19 ≤ h ≤ 19, -9 ≤ k ≤ 9, -10 ≤ l ≤ 9	-18 ≤ h ≤ 17, -18 ≤ k ≤ 17, 0 ≤ l ≤ 19
Reflections collected	9048	6523
Independent reflections	1056 [R_{int} = 0.0529, R_{sigma} = 0.0276]	6470 [R_{int} = 0.0089, R_{sigma} = 0.0160]
Data/restraints/parameters	1056/0/49	6470/1/405
Goodness-of-fit on F^2	1.054	1.093
Final R indexes [I>=2σ (I)]	R_1 = 0.0257, wR_2 =	R_1 = 0.0387,

	0.0589	wR_2 = 0.0997
**Final R indexes [all data]**	R_1 = 0.0322, wR_2 = 0.0641	R_1 = 0.0400, wR_2 = 0.1002
**Largest diff. peak/hole / e Å^{-3}**	0.48/-0.40	0.36/-0.27
**Flack parameter**	-	0.51(9)
**CCDC number**	2223424	2223431

**Compound**	**[2,6-lutidine)$_2$Cl][Cl$_3$]**	**[Ag(py)$_4$][BF$_4$]$_2$**	**[Cl$_2$F][AsF$_6$]**	**[C$_5$F$_5$NCl][AsF$_6$]**
**Identification code**	P21_m_final	Pbcn_notwin	P21_n	P21_n_twin
**Empirical formula**	$C_{14}H_{18}Cl_4N_2$	$C_{26}H_{30}AgB_2F_8N_6$	$AsCl_2F_7$	$C_5AsClF_{11}N$
**Formula weight**	356.10	708.05	278.82	393.43
**Temperature/K**	100.0	100.0	100.0	103.0
**Crystal system**	monoclinic	orthorhombic	monoclinic	monoclinic
**Space group**	$P2_1/m$	*Pbcn*	$P2_1/n$	$P2_1/n$
***a*/Å**	8.3507(10)	13.6718(18)	5.3882(7)	6.2789(8)
***b*/Å**	6.7026(6)	15.798(2)	15.2962(17)	21.884(2)
***c*/Å**	15.1667(17)	15.142(2)	7.9427(10)	7.7197(9)
***α*/°**	90	90	90	90
***β*/°**	104.077(5)	90	105.459(4)	111.310(4)
***γ*/°**	90	90	90	90
**Volume/Å^3**	823.41(15)	3270.6(7)	630.95(13)	988.2(2)
**Z**	2	4	4	4
**ρ_{calc}g/cm^3**	1.436	1.438	2.935	2.644
**μ/mm^{-1}**	0.710	0.687	6.299	3.862
**F(000)**	368.0	1428.0	520.0	744.0
**Crystal size/mm^3**	0.35 × 0.12 × 0.07	0.33 × 0.3 × 0.29	0.25 × 0.23 × 0.19	0.6 × 0.31 × 0.22
**Radiation**	MoK$_\alpha$ (λ = 0.71073)	MoK$_\alpha$ (λ = 0.71073)	MoK$_\alpha$ (λ = 0.71073)	MoK$_\alpha$ (λ = 0.71073)
**2Θ range for data**	5.118 to 56.708	3.94 to 52.846	5.326 to	5.962 to 52.832

**collection/°**			52.856	
**Index ranges**	-9 ≤ h ≤ 11, -8 ≤ k ≤ 8, -20 ≤ l ≤ 20	-14 ≤ h ≤ 17, -19 ≤ k ≤ 19, -18 ≤ l ≤ 18	-6 ≤ h ≤ 6, -19 ≤ k ≤ 19, -9 ≤ l ≤ 9	-7 ≤ h ≤ 7, -27 ≤ k ≤ 0, -9 ≤ l ≤ 9
**Reflections collected**	7667	23765	31194	3943
**Independent reflections**	2197 [R_{int} = 0.0280, R_{sigma} = 0.0274]	3359 [R_{int} = 0.0351, R_{sigma} = 0.0227]	1297 [R_{int} = 0.0490, R_{sigma} = 0.0142]	2013 [R_{int} = 0.0945, R_{sigma} = 0.0922]
**Data/restraints/parameters**	2197/0/125	3359/0/207	1297/0/91	2013/0/172
**Goodness-of-fit on F^2**	1.059	1.247	1.221	1.095
**Final R indexes [I>=2σ (I)]**	R_1 = 0.0250, wR_2 = 0.0662	R_1 = 0.0539, wR_2 = 0.1249	R_1 = 0.0243, wR_2 = 0.0628	R_1 = 0.0425, wR_2 = 0.0957
**Final R indexes [all data]**	R_1 = 0.0287, wR_2 = 0.0680	R_1 = 0.0589, wR_2 = 0.1274	R_1 = 0.0244, wR_2 = 0.0629	R_1 = 0.0548, wR_2 = 0.0991
**Largest diff. peak/hole / e $Å^{-3}$**	0.41/-0.24	1.59/-0.49	0.60/-0.87	1.23/-0.76
**Flack parameter**	-	-	-	-
**CCDC number**	2223423	2223425	2223439	2223421

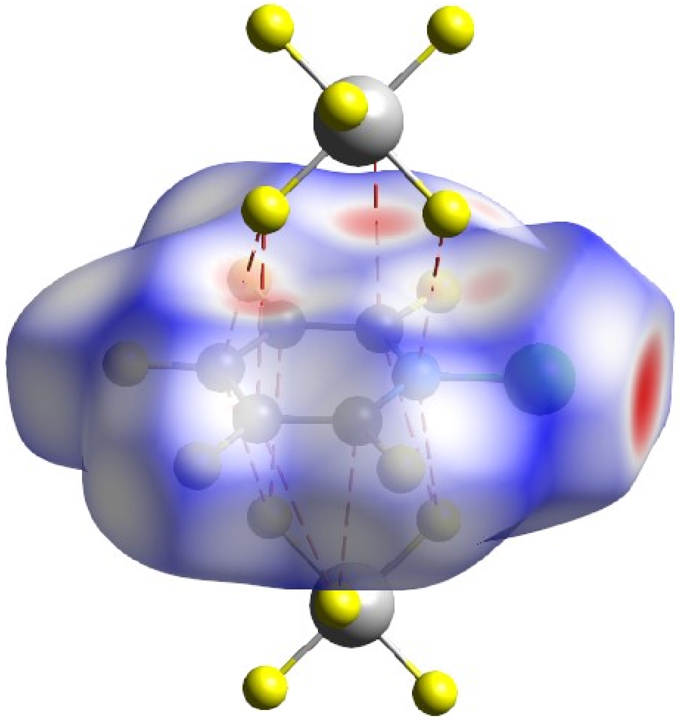

Figure S1. Hirshfeld surface of $[C_5F_5NCl]^+$ with two $[AsF_6]^-$ anions above and below the ring plane. Dashed lines indicate close contacts between fluorine atoms of the anions and the carbon or nitrogen atom of the ring.

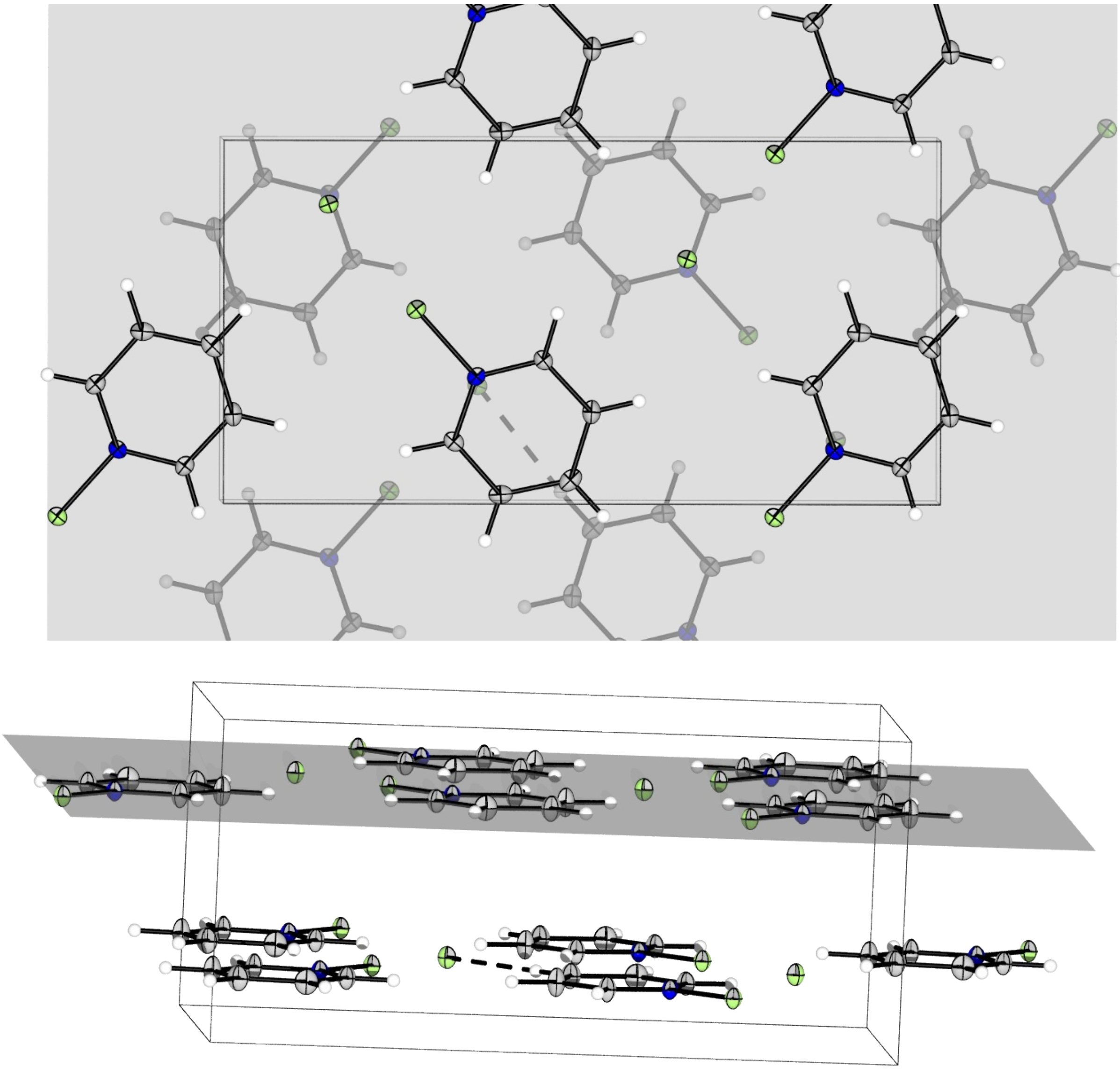

Figure S2. Solid-state structure (top: top view, bottom: side view) of [pyCl]Cl with cell edges and a plane illustrating the layered structure of the compound. Color code: green = chlorine, blue = nitrogen, grey = carbon, white = hydrogen. Dashed line illustrates a hydrogen bond between the negatively polarized chlorine atom Cl2 and a H atom in 4-position.

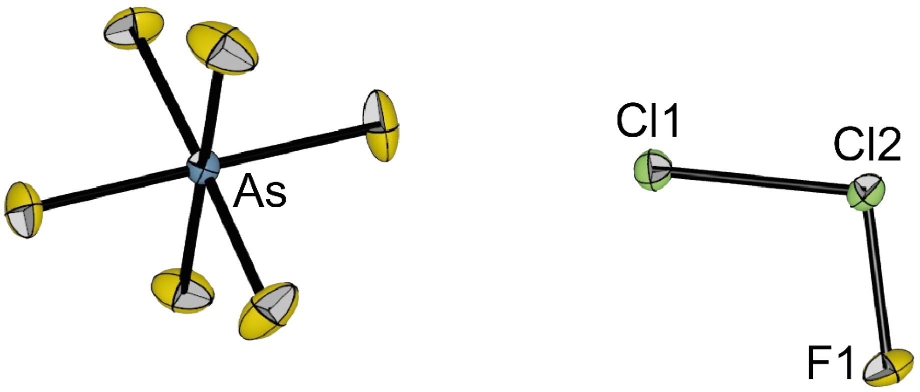

Figure S3. Solid-state structure of $[Cl_2F][AsF_6]$. Color code: green = chlorine, yellow = fluorine, light blue = arsenic. Selected bond distances (pm) and angles (°): Cl1-Cl2 193.87(9), Cl2-F1 159.4(2), Cl1-Cl2-F1 101.83(7).

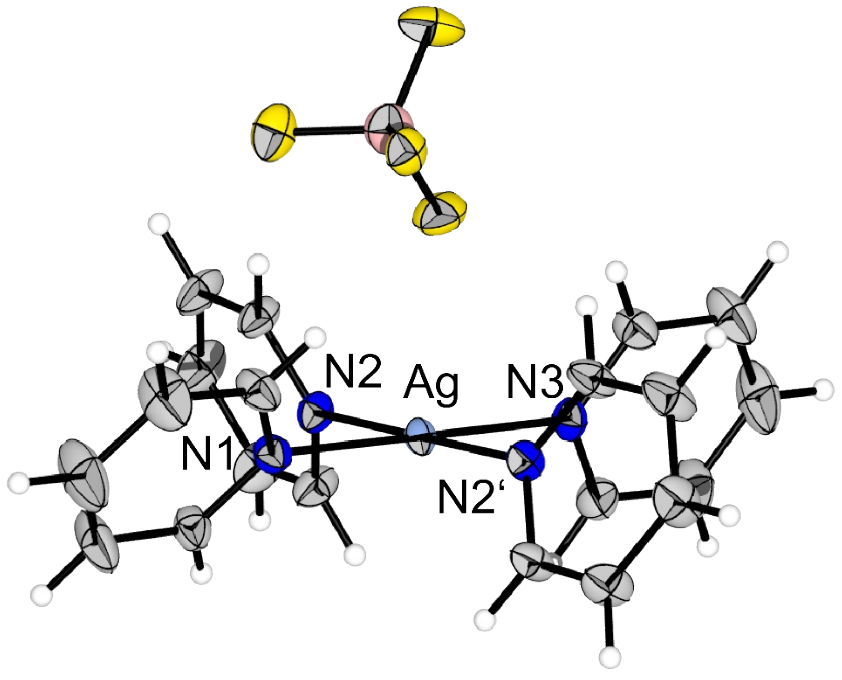

Figure S4. Solid-state structure of $[Ag(py)_4][BF_4]$. Solvent omitted for clarity. Silver atom lies on symmetry element therefore only one $[BF_4]^-$ counterion is visible. Color code: light blue = silver, blue = nitrogen, grey = carbon, white = hydrogen, yellow = fluorine, rose = boron. Selected bond distances (pm): Ag-N1 216.8(5), Ag-N2 216.8(3), Ag-N3 219.0(5).

Spectra

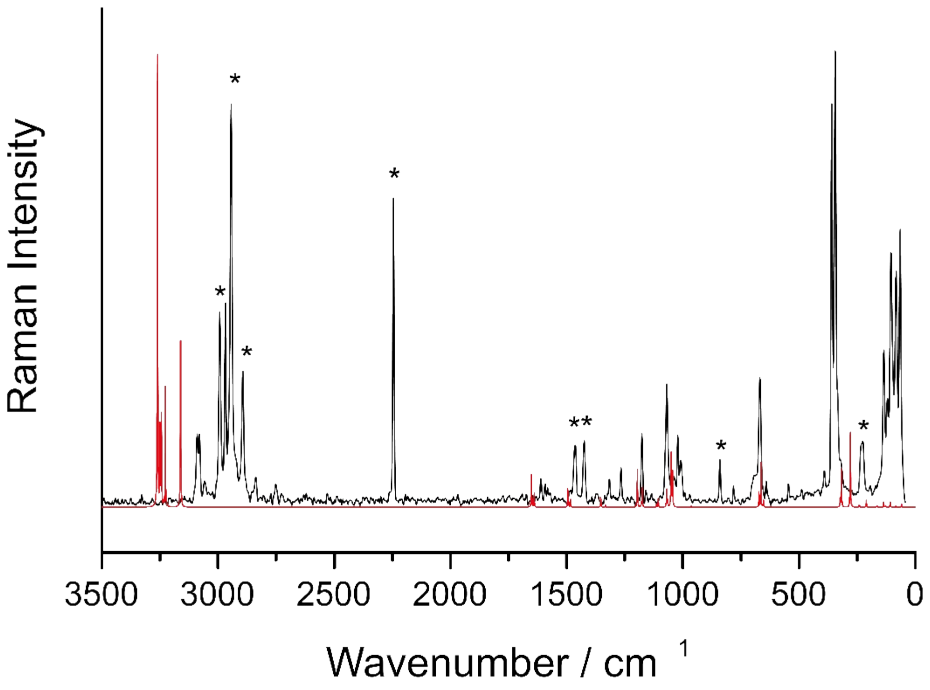

Figure S5. Raman spectrum (1064 nm, 77 K, crystal) of [pyCl]Cl. Experimental spectrum (black), calculated spectrum (red, solid-state calculation, see computational details). Asterisk denotes residual solvent bands.

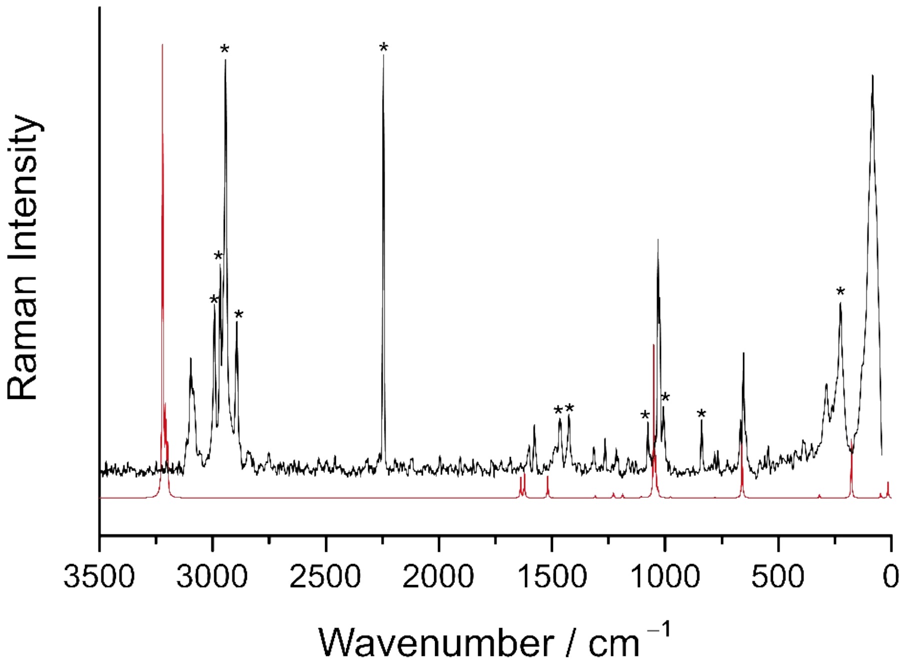

Figure S6. Raman spectrum (1064 nm, 77 K, solid) of [py_2Cl][BF_4]. Experimental spectrum (black), calculated spectrum (red, B3LYP/def2-TZVPP). Asterisk denotes residual solvent bands.

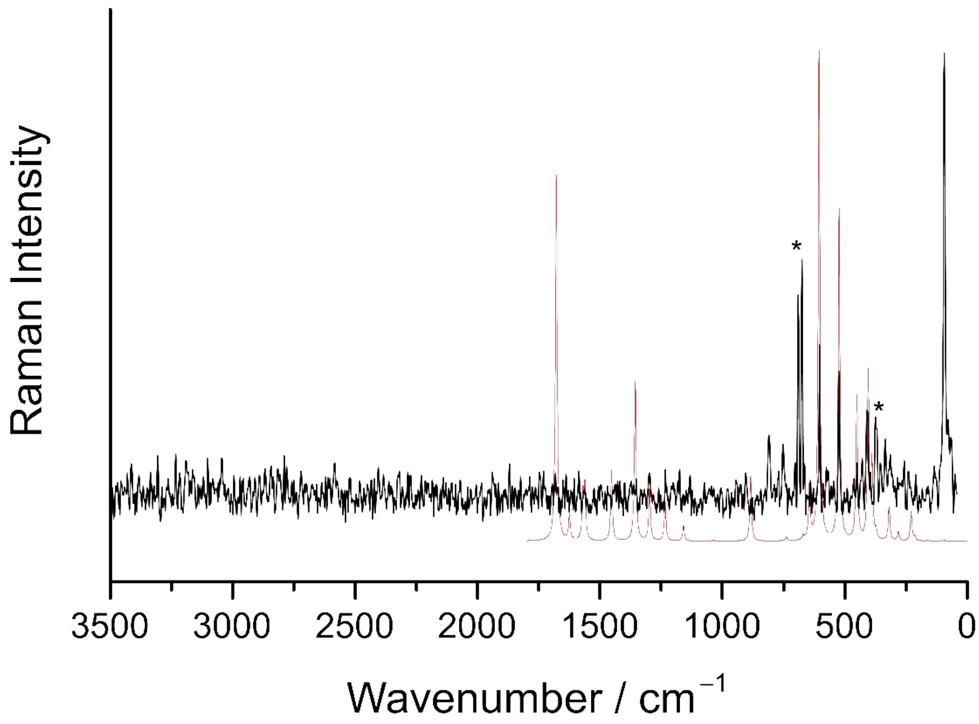

Figure S7. Raman spectrum (1064 nm, 77 K, solid) of [C_5F_5NCl][AsF_6]. Experimental spectrum (black), calculated spectrum (red, B3LYP/def2-TZVPP). Asterisk denotes anion bands.

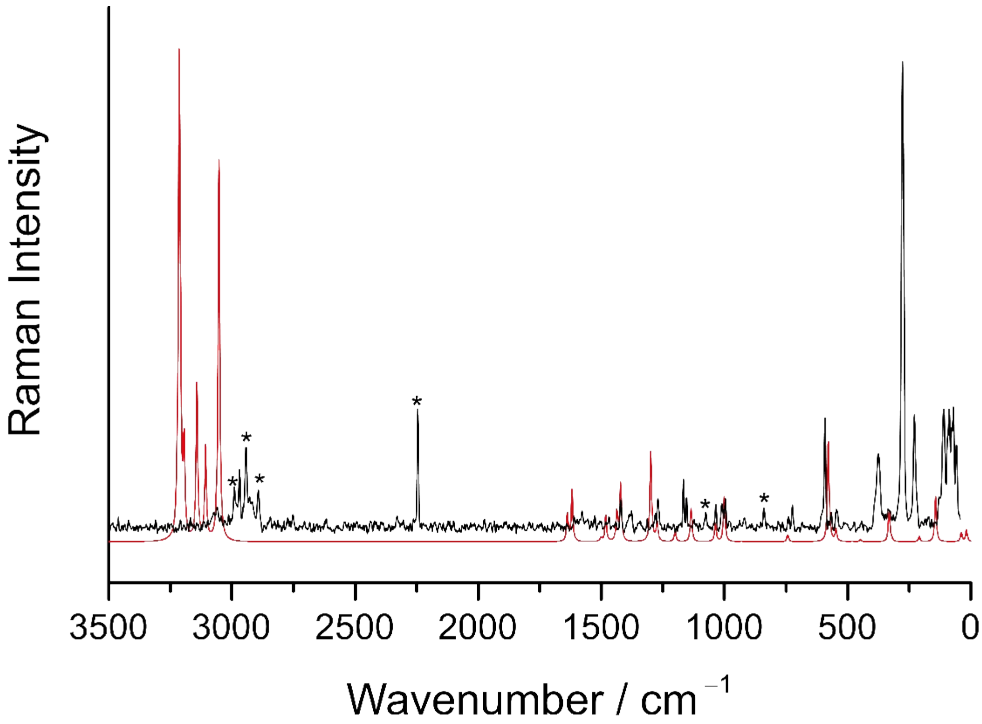

Figure S8. Raman spectrum (1064 nm, 77 K, solid) of [(2,6-lutidine)$_2$Cl][Cl_3]. Experimental spectrum (black), calculated spectrum (red, B3LYP/def2-TZVPP). Asterisk denotes solvent bands.

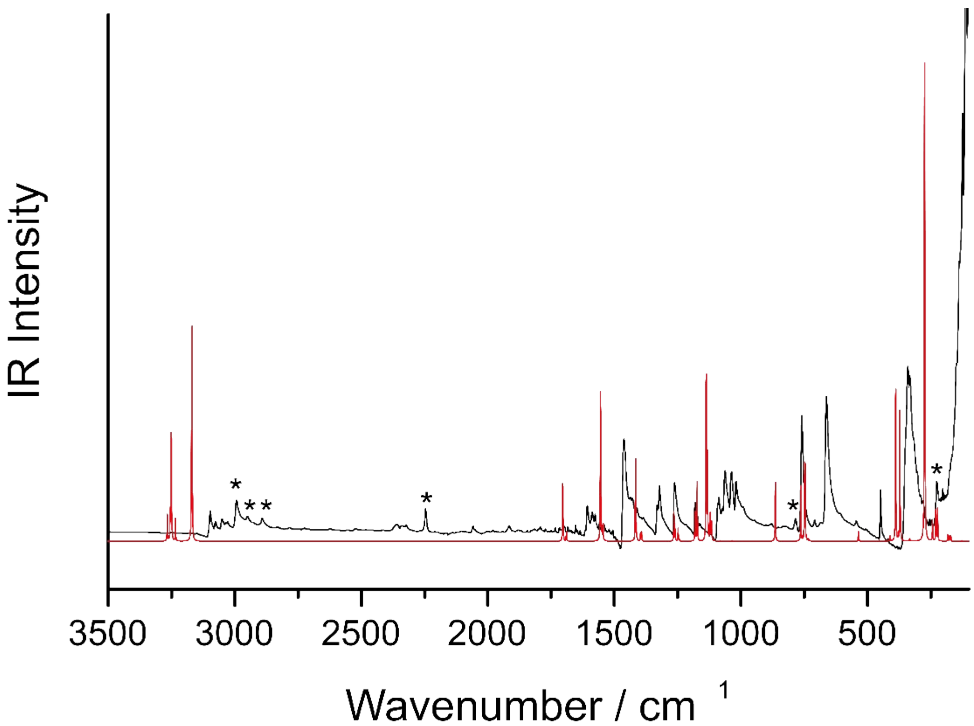

Figure S9. IR Spectrum (ATR, 193 K, solid) of [pyCl]Cl. Experimental spectrum (black), calculated spectrum (red, solid-state calculation, see computational details). Asterisk denotes residual solvent bands.

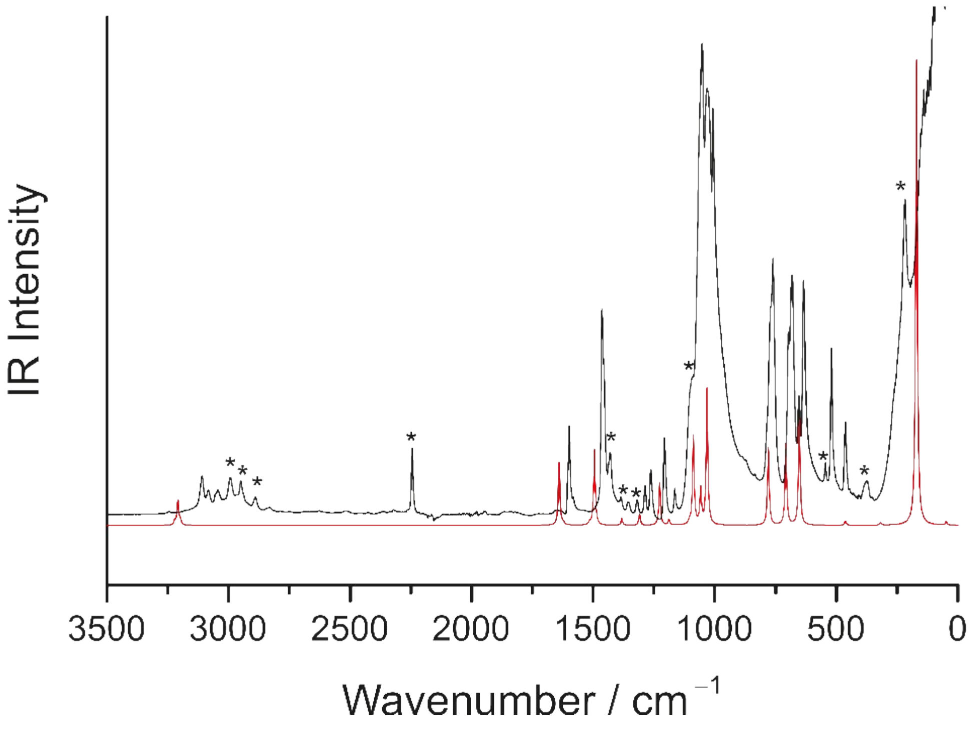

Figure S10. IR Spectrum (ATR, 193 K, solid) of [py_2Cl][BF_4]. Experimental spectrum (black), calculated spectrum (red, B3LYP/def2-TZVPP). Asterisk denotes residual solvent bands.

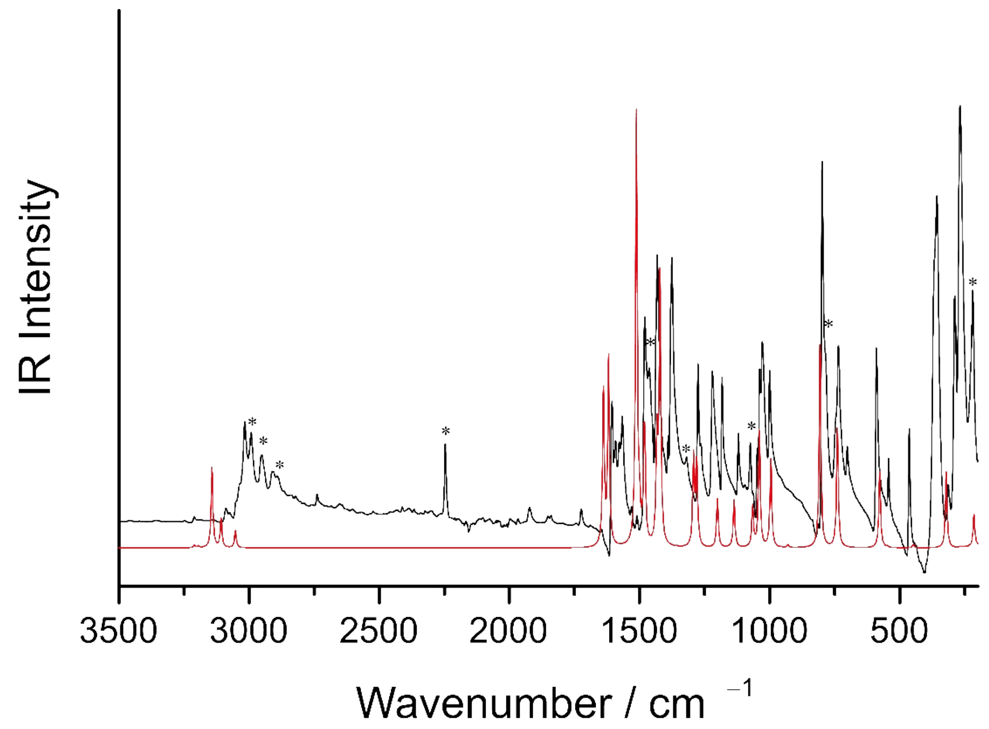

Figure S11. IR Spectrum (ATR, 193 K, solid) of [(2,6-lutidine)$_2$Cl][Cl$_3$]. Experimental spectrum (black), calculated spectrum (red, B3LYP/def2-TZVPP). Asterisk denotes solvent bands.

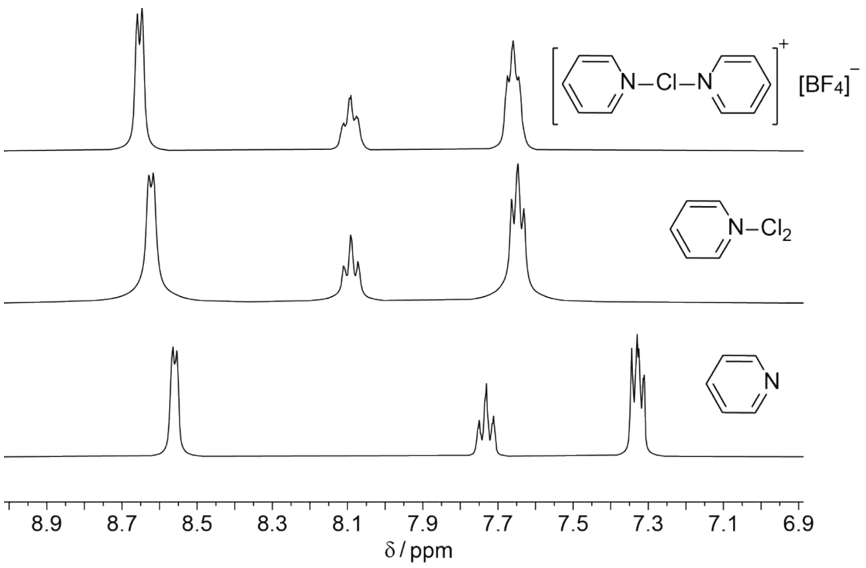

Figure S12. ^{1}H NMR spectra (400 MHz, MeCN-d3, 253 K) of pyridine (bottom), py·Cl$_2$ (middle) and [py$_2$Cl][BF$_4$] (top).

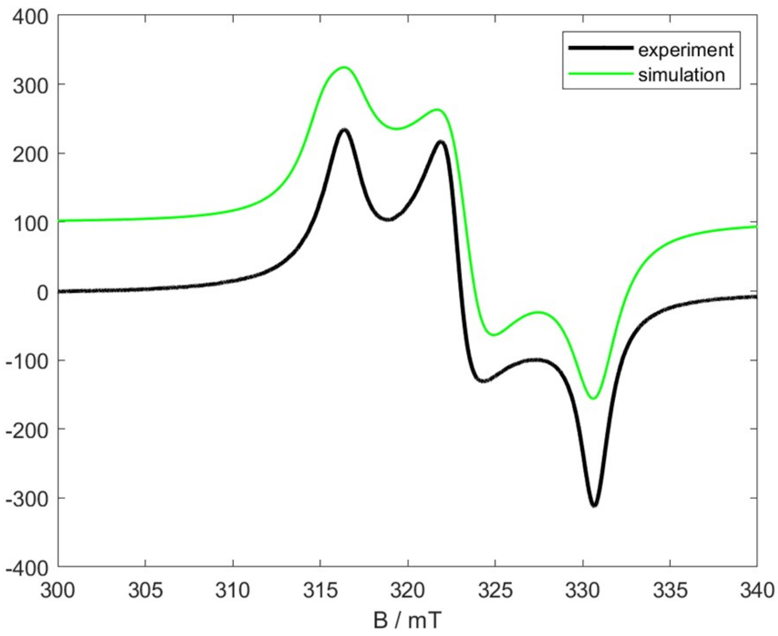

Figure S13. EPR spectrum (solid, X-band, 298 K) of $[Ag(py)_4][BF_4]_2$.Experimental spectrum (black) and simulated spectrum (green). The measured g tensors from its solid-state EPR (Figure Sx) spectrum are gx = 2.035, gy = 2.081, gz = 2.130. They deviate slightly from literature known values of $[Ag(py)_4][S_2O_8]$ due to the different counter ion.[17]

Computational section:

Xyz coordinates of $[pyCl]^+$

N	-0.000000	0.000001	1.268225
Cl	-0.000000	0.000028	2.977644
C	1.187022	-0.000006	0.627595
C	1.201157	-0.000002	-0.748868
C	-0.000000	0.000013	-1.448054
C	-1.201157	-0.000003	-0.748868
C	-1.187022	-0.000006	0.627595
H	2.071985	-0.000014	1.242199
H	2.153624	-0.000013	-1.255809
H	0.000000	0.000028	-2.528046
H	-2.153624	-0.000015	-1.255809
H	-2.071985	-0.000012	1.242199

Xyz coordinates of $[py_2Cl]^+$

N	-1.999751	0.102844	-0.000093
Cl	-0.000010	0.000001	-0.000140
N	1.999751	-0.102843	-0.000134
C	2.709387	1.027522	-0.000038
C	4.093079	0.990881	0.000072
C	4.732115	-0.243364	0.000093
C	3.969835	-1.405542	0.000010
C	2.589685	-1.300043	-0.000103
C	-2.589684	1.300044	-0.000070
C	-3.969835	1.405542	0.000015

C	-4.732114	0.243364	0.000076
C	-4.093078	-0.990881	0.000068
C	-2.709386	-1.027522	-0.000014
H	-1.938644	2.162604	-0.000126
H	-4.430736	2.382412	0.000039
H	-5.811834	0.298892	0.000119
H	-4.651760	-1.915316	0.000127
H	-2.150266	-1.952318	-0.000018
H	1.938645	-2.162603	-0.000177
H	2.150269	1.952318	-0.000052
H	4.651761	1.915316	0.000144
H	5.811835	-0.298892	0.000162
H	4.430736	-2.382413	0.000040

Xyz coordinates of $[(2,6\text{-lutidine})_2Cl]^+$

C	2.676626	-0.080880	1.184746
N	2.031259	-0.101949	0.001409
C	2.672055	-0.188465	-1.181438
C	4.060272	-0.259621	-1.192311
C	4.761084	-0.240861	0.002451
C	4.064887	-0.151008	1.196679
Cl	0.000070	0.001365	0.000635
N	-2.031122	0.104672	-0.000119
C	-2.734326	-1.044362	0.049416
C	-4.123146	-0.985143	0.049449
C	-4.760950	0.243517	-0.001004
C	-4.001743	1.401122	-0.051007
C	-2.614081	1.319123	-0.050072
C	-1.760609	2.548788	-0.103324
H	-4.470265	2.373439	-0.091007
H	-5.841002	0.298451	-0.001352
H	-4.687929	-1.904904	0.089108
C	-2.010057	-2.354275	0.103157
C	1.890210	0.017023	2.455687
C	1.880731	-0.206075	-2.452982
H	4.575095	-0.328967	-2.139233
H	5.841135	-0.295822	0.002863
H	4.583368	-0.134241	2.143996
H	-2.723601	-3.173138	0.138913
H	-1.373552	-2.487632	-0.771609
H	-1.370269	-2.413819	0.983647
H	-2.387364	3.435822	-0.139500
H	-1.114397	2.616907	0.771837
H	-1.117539	2.543076	-0.983419
H	2.549234	-0.278913	-3.306655
H	1.194657	-1.052746	-2.477254
H	1.283768	0.699992	-2.556992
H	2.562007	0.021711	3.309870
H	1.293570	0.928941	2.479444
H	1.204312	-0.823787	2.559237

Xyz coordinates of $[C_5F_5NCl]^+$

N	0.055055	0.000012	-0.024089
C	0.017781	0.000010	1.332718
C	1.187710	-0.000005	2.061321
C	2.406420	-0.000015	1.387628
C	2.428124	-0.000004	-0.004732
C	1.235081	0.000011	-0.694821
F	-1.146682	0.000027	1.899558
F	1.140777	0.000004	3.375008
F	3.524563	-0.000022	2.058929
F	3.565553	0.000006	-0.663695
F	1.187963	0.000029	-1.989064
Cl	-1.402149	-0.000050	-0.898971

Optimized parameters of [pyCl]Cl

Optimized cell parameter: 13.9435 6.5593 7.0669

Optimized fractional coordinates of the asymmetric unit cell:

17	-1.397546578618E-01	-2.500000000000E-01	1.924339192579E-01
17	-2.531036418042E-01	-2.500000000000E-01	4.477663575600E-01
7	-3.428151408074E-01	-2.500000000000E-01	-3.558099252295E-01
6	-4.314986439129E-01	-2.500000000000E-01	-4.021759422905E-01
1	-4.486048301271E-01	-2.500000000000E-01	4.504484463959E-01
6	-3.783116024211E-01	-2.500000000000E-01	-3.512558800079E-02
1	-3.531448186011E-01	-2.500000000000E-01	1.074387043148E-01
6	-3.147161079357E-01	-2.500000000000E-01	-1.784536852871E-01
1	-2.416065520397E-01	-2.500000000000E-01	-1.516163325766E-01
6	-4.712451304556E-01	-2.500000000000E-01	-7.766980163571E-02
1	4.775869300957E-01	-2.500000000000E-01	3.306440635615E-02
6	-4.977914041016E-01	-2.500000000000E-01	-2.642781954815E-01
1	4.308142847581E-01	-2.500000000000E-01	-3.063822769933E-01

References:

References

1 O. Ruff, E. Ascher, J. Fischer and F. Laass, *Z. anorg. allg. Chem.*, 1928, **176**, 258.
2 P. Voßnacker, S. Steinhauer, J. Bader and S. Riedel, *Chem. Eur. J.*, 2020, **26**, 13256.
3 P. Pröhm, W. R. Berg, S. M. Rupf, P. Voßnacker and S. Riedel, *Chem. Eur. J.*, 2021, **27**, 17676.
4 R. K. Harris, E. D. Becker, S. M. Cabral de Menezes, P. Granger, R. E. Hoffman and K. W. Zilm, *Pure Appl. Chem.*, 2008, **80**, 59.
5 G. M. Sheldrick, *Acta Cryst. A*, 2015, **71**, 3.
6 G. M. Sheldrick, *Acta Cryst. C,* 2015, **71**, 3.
7 O. V. Dolomanov, L. J. Bourhis, R. J. Gildea, J. A. K. Howard and H. Puschmann, *J. Appl. Cryst.*, 2009, **42**, 339.
8 K. Brandenburg, *DIAMOND* (3.2 ed), Crystal Impact GbR, Bonn, 2014.
9 M. J. Frisch, G. W. Trucks, H. B. Schlegel, G. E. Scuseria, M. A. Robb, J. R. Cheeseman, G. Scalmani, V. Barone, G. A. Petersson, H. Nakatsuji, X. Li, M. Caricato, A. V. Marenich, J. Bloino, B. G. Janesko, R. Gomperts, B. Mennucci, H. P. Hratchian, J. V. Ortiz, A. F. Izmaylov, J. L. Sonnenberg, D. Williams-Young, F. Ding, F. Lipparini, F. Egidi, J. Goings, B. Peng, A. Petrone, T. Henderson, D. Ranasinghe, V. G. Zakrzewski, J. Gao, N. Rega, G. Zheng, W. Liang, M. Hada, M. Ehara, K. Toyota, R. Fukuda, J. Hasegawa, M. Ishida, T. Nakajima, Y. Honda, O. Kitao, H. Nakai, T. Vreven, K. Throssell, J. A. Montgomery, Jr., J. E. Peralta, F. Ogliaro, M. J. Bearpark, J. J. Heyd, E. N. Brothers, K. N. Kudin, V. N. Staroverov, T. A. Keith, R. Kobayashi, J. Normand, K. Raghavachari, A. P. Rendell, J. C. Burant, S. S. Iyengar, J. Tomasi, M. Cossi, J. M. Millam, M. Klene, C. Adamo, R. Cammi, J. W. Ochterski, R. L. Martin, K. Morokuma, O. Farkas, J. B. Foresman, and D. J. Fox, Gaussian 16 (Revision A.03), Gaussian, Inc., Wallingford CT, 2016.
10 C. Lee, W. Yang and R. G. Parr, *Phys. Rev. B*, 1988, **37**, 785.
11 F. Weigend and R. Ahlrichs, *Phys. Chem. Chem. Phys.*, 2005, **7**, 3297.
12 a) S. Miertuš, E. Scrocco and J. Tomasi, *Chem. Phys.*, 1981, **55**, 117; b) S. Miertuš̃ and J. Tomasi, *Chem. Phys.*, 1982, **65**, 239; c) J. L. Pascual-ahuir, E. Silla and I. Tuñon, *J. Comput. Chem.*, 1994, **15**, 1127;
13 E. D. Glendening, J. K. Badenhoop, A. E. Reed, J. E. Carpenter, J. A. Bohmann, C. M. Morales, P. Karafiloglou, C. R. Landis and F. Weinhold, NBO 7.0 (7.0.4), Theoretical Chemistry Institute, University of Wisconsin, Madison, WI, 2018.
14 R. Dovesi, A. Erba, R. Orlando, C. M. Zicovich-Wilson, B. Civalleri, L. Maschio, M. Rérat, S. Casassa, J. Baima, S. Salustro and B. Kirtman, *WIREs Comput Mol Sci*, 2018, **8**, e1360.
15 a) T. H. Dunning, *J. Chem. Phys.*, 1989, **90**, 1007; b) D. E. Woon and T. H. Dunning, *J. Chem. Phys.*, 1993, **98**, 1358;
16 A. Karim, M. Reitti, A.-C. C. Carlsson, J. Gräfenstein and M. Erdélyi, *Chem. Sci.*, 2014, **5**, 3226.
17 J. A. McMillan and B. Smaller, *J. Inorg. Nucl. Chem.*, 1961, **35**, 1698.
18 R. J. Gillespie and M. J. Morton, *Inorg. Chem.*, 1970, **9**, 811.
